# Four new species of Acarosporaceae (Acarosporales, Lecanoromycetes) with carbonized epihymenial accretions from China

**DOI:** 10.3897/mycokeys.133.196437

**Published:** 2026-06-08

**Authors:** Fu Hui Liang, Jia Xin Wang, Min Ai, Wei Jiang, Shu Nuo Zhou, Zun Tian Zhao, Xin Yu Wang, Ling Hu

**Affiliations:** 1 College of Geography and Environment, Shandong Normal University, Jinan 250300, China College of Geography and Environment, Shandong Normal University Jinan China; 2 College of Life Sciences, Shandong Normal University, Jinan 250300, China College of Life Sciences, Shandong Normal University Jinan China; 3 State Key Laboratory of Phytochemistry and Natural Medicines, Kunming Institute of Botany, Chinese Academy of Sciences, 650201 Kunming, China State Key Laboratory of Phytochemistry and Natural Medicines, Kunming Institute of Botany, Chinese Academy of Sciences Kunming China; 4 Yunnan Key Laboratory for Fungal Diversity and Green Development, Kunming Institute of Botany, Chinese Academy of Sciences, 650201 Kunming, China Yunnan Key Laboratory for Fungal Diversity and Green Development, Kunming Institute of Botany, Chinese Academy of Sciences Kunming China; 5 Jiangsu Changhuan Environmental Technology Co., Ltd., Changzhou, China School of Environment, Nanjing Normal University Nanjing China; 6 School of Environment, Nanjing Normal University, No.1 Wenyuan Rd, Nanjing 210023, China Jiangsu Changhuan Environmental Technology Co., Ltd. Changzhou China; 7 Yantai Kunyu Mountain Forest Farm, Yantai, 264112, China Yantai Kunyu Mountain Forest Farm Yantai China

**Keywords:** Carbonized margin, gyrose disc, phylogeny, *
Polysporina
*, taxonomy

## Abstract

Based on a combination of morphological, chemical, and phylogenetic analyses, we report four new species from China: *Acarospora carbonacea*, *A. rorida*, *Sarcogyne knudsenii*, and *S. xizangensis*. These four species are characterized by endolithic thalli, apothecia with carbonized epihymenial accretions, an *Acarospora*-type ascus, and the absence of secondary metabolites. *Acarospora carbonacea* sometimes can develop a white epilithic ecorticate thallus, whereas such a thallus is not observed in the other three species. Comprehensive descriptions, detailed illustrations, and phylogenetic analyses of the four new species are provided. In addition, a compiled species checklist summarizing the diagnostic features of the eight reported species of Acarosporaceae with carbonized epihymenial accretions in China is presented.

## Introduction

The genus *Sarcogyne* Flot. was validly named and published through the publication of Flotow’s letters (Flotow 1850, 1851; Knudsen et al. 2023a), however, he did not publish the type species and provide a circumscription of the genus. It was subsequently defined by Massalongo (1854) and Körber (1855) as comprising species with lecideine apothecia, disc surface with or without carbonized accretions, multispored asci (more than 100 ascospores per ascus), and a non-amyloid tholus. Magnusson (1929, 1935a, 1935b) maintained these morphological genus concepts of *Sarcogyne* in the family Acarosporaceae Zahlbr. Knudsen et al. (2021a) proposed to conserve *Sarcogyne clavus* as the type of the genus.

The lichenologist A. Vězda (1978) established the new genus *Polysporina* Vězda to include those lecideine species of *Sarcogyne* with carbonized epihymenium accretions on the disc surface. Consequently, the morphological distinction between the two genera is simply that the apothecia of *Polysporina* develop carbonized epihymenium accretions (Seppelt et al. 1998; Knudsen and Etayo 2009; Knudsen and Kocourková 2009, 2011, 2018a).

Through molecular phylogenetic studies based on the combination of four gene loci (ITS, nuLSU, mtSSU and β-tubulin), Westberg et al. (2015) revealed that *Polysporina* is a polyphyletic genus, and carbonization seems to be a derived character that has appeared during particular episodes in the evolution of the Acarosporomycetidae/Acarosporaceae. The type species *Polysporina simplex* nom. illegit. (=*Acarospora privigna*) as well as other taxa with carbonized epihymenial accretions, such as *A. leavitti* and *A. subfuscescens* were recovered within a non-monophyletic *Acarospora* group (Knudsen et al. 2021a, 2021b). In addition, several *Polysporina* taxa are currently being treated as *Sarcogyne*, such as *S. cyclocarpa*, *S. urceolata* and *S. pusilla* (Knudsen et al. 2013, 2021b; Tsurykau et al. 2024). Therefore, species with lecideine apothecia can occur in both groups as well as species with carbonized epihymenial accretions (Westberg et al. 2015; Knudsen et al. 2016, 2021b).

According to the research of Westberg et al. (2015) and Knudsen et al. (2023a, 2023b, 2025a), the genus *Polysporina* has been dissolved, and the remaining species can only be placed in either the *Acarospora* or *Sarcogyne* groups through phylogenetic analysis, rather than through morphological and anatomical characteristics.

Before this study, four species with carbonized epihymenium accretions have been reported in China: *Acarospora privigna* (=*Polysporina simplex*), *A. subfuscescens* (≡*Sarcogyne sinensis*), *Sarcogyne cyclocarpa* (=*Polysporina cyclocarpa*), and *S. gyrocarpa* (Magnusson 1940; Aptroot and Seaward 1999; Aptroot and Sparrius 2003). We continue the study of species with carbonized apothecia in the Acarosporaceae from China and have collected specimens from five provinces: Shandong, Jiangsu, Gansu, Xizang and Xinjiang. These species are characterized by an endolithic thallus, carbonized ascomata, and discs with dark brown or black epihymenial accretions on the surface. Based on integrated morphology and molecular analyses, four new species were identified and named *Acarospora carbonacea*, *A. rorida*, *Sarcogyne knudsenii* and *S. xizangensis*. Below, these new species are described in detail, and a checklist summarizing the diagnostic features of eight species of Acarosporaceae with carbonized epihymenial accretions from China is provided (Table 1).

**Table 1.** Morphological Characteristics’ list of Acarosporaceae with carbonized epihymenial accretions in China.

Species	Hymenium (μm)	Hymenium IKI reaction	Paraphyses (μm)	Asci (μm)	Ascospores (μm)	Substrate	References	Note
*Acarospora carbonacea*	60–90	red or blue turning red, hemiamyloid	1–2	60–80 × 20–25	4–5 × 2–3	siliceous rock	This paper	-
*A. rorida*	50–125	reddish orange, rarely light blue turning reddish orange, hemiamyloid	1–2	50–100 × 12–20	3–4 × 1–2	calcareous rock	This paper	-
*A. privigna*	(60–) 90–130	light blue fading to light red, hemiamyloid	1–1.5	(55–) 65–100 (–110) × (12–) 15–17 (–20)	3–5 (–5.5) × 1–1.5	siliceous or weakly calcareous rocks	Aptroot and Sparrius (2003); Knudsen et al. (2021b)	=*Polysporina simplex*
*A. subfuscescens*	(80–)100–140(–180)	blue turning red, but often reaction various from red to yellow-green, hemiamyloid	1–2(–3)	50–110 × 12–20	(3–)4–4.5(–5) × (1–)1.5–2(–3)	parasite	Magnusson (1940); Knudsen et al. (2021b)	≡*Sarcogyne sinensis*
*Sarcogyne cyclocarpa*	(60–)80–90 (–110)	usually immediately red, rarely blue to red, hemiamyloid	1–2	40–60 × 15–20	(3.5–)4–5(–6) × (1–)2(–2.5)	restricted to calcareous rocks	Aptroot and Seaward (1999); Knudsen et al. (2021b)	=*Polysporina cyclocarpa*
*S. gyrocarpa*	(80–)100–140	blue to yellow-green, or blue turning to red or smoky brown to yellow-green, hemiamyloid	(1.5–)2–3	70–90 × 18–25	(3.5–)4–5(–7) × (2–)2.5–3(–4)	siliceous or calcareous rock	Magnusson (1940); Knudsen et al. (2021c)	-
*S. knudsenii*	50–100	blue, euamyloid	2	60–75 × 15–20	6–7 × 3–4	calcareous rock	This paper	-
*S. xizangensis*	60–95	red or blue turning red, hemiamyloid	2.5–4	50–70 × 12–20	4–6 × 2–3	siliceous or calcareous rock	This paper	-

## Material and methods

### Morphological and chemical analyses

Specimens were collected from the provinces of Shandong, Jiangsu, Gansu, Xizang and Xinjiang Uygur Autonomous Region, and are preserved in the herbarium of the Kunming Institute of Botany, Chinese Academy of Sciences (KUN) and in the Lichen Section of the Botanical Herbarium at Shandong Normal University, Jinan, China (SDNU). Morphology characters were observed under a dissecting microscope (COIC XTL7045B2), and the anatomy characters were examined and measured under an optical microscope (OLYMPUS CX21). Photos were taken with a digital camera (DP72) along with a stereomicroscope (OLYMPUS SZX16) and a compound microscope (OLYMPUS BX61). Secondary metabolites were identified and analyzed by thin-layer chromatography (TLC) using solvents C (toluene: acetic acid = 170:30; Orange et al. 2010). The amyloid reaction of lichen specimens hymenial gel and subhymenium was tested with fresh undiluted IKI (Lugol’s iodine solution, 1%; Knudsen and Kocourková 2018b).

### DNA extraction, PCR amplification and sequencing

For molecular analysis, genomic DNA was extracted from the thalli and apothecia of dried lichen specimens using the Sigma-Aldrich REDExtract-N-Amp Plant PCR Kit (St. Louis, MO, USA), following the instructions of the manufacturer. The internal transcribed spacer region (ITS), nuclear large subunit rDNA (nuLSU), mitochondrial small subunit rDNA (mtSSU) and the protein encoding sequence of the β-tubulin gene were respectively amplified using the primer pair ITS1F/ITS4 (White et al. 1990; Gardes and Bruns 1993), LR0R/LR5 (Vilgalys and Hester 1990; Rehner and Samuels 1995), mtSSU1/mtSSU3R (Zoller et al. 1999), Bt3/Bt10 (β-TUB; Einax and Voigt 2003). Standard PCR reactions were performed in a total volume of 25 μL, each reaction contained 12.5 μL of MasterMix (Tiangen, Beijing, China), 8.5 μL of ddH_2_O, 1 μL of upstream primer, 1 μL of downstream primer, and 2 μL of DNA extract. The PCR conditions for nuLSU: initial denaturation at 98 °C for 3 min, followed by 30 cycles (98 °C for 10s, 56 °C for 10s, and 72 °C for 15s), with a final extension at 72 °C for 10 min (Wang et al. 2025). The PCR conditions for ITS and mtSSU: initial denaturation at 95 °C for 5 min, followed by five cycles (95 °C for 33 s, 56 °C for 30 s, and 72 °C for 30 s), then ten cycles (95 °C for 30 s, 54 °C for 30 s, and 72 °C for 30 s), and twenty cycles (95 °C for 30 s, 50 °C for 30 s, and 72 °C for 30 s) with a final extension at 72 °C for 15 min (Knudsen et al. 2025b). The PCR conditions for β-TUB: initial denaturation 95 °C for 1 min, followed by five cycles (95 °C for 30 s, 60 °C for 30 s, and 72 °C for 60 s), and finally 30 cycles (95 °C for 15 s, 55 °C for 30 s, and 72 °C for 45 s), with a final extension 72 °C for 10 min (Knudsen et al. 2025a). The polymerase chain reaction (PCR) products were sequenced by Sangon Biotech (Jinan, China).

### Phylogenetic analyses

A BLAST search was performed to identify similar sequences in GenBank. Raw DNA sequences were assembled and edited using Geneious Prime, and sequences of related species and the outgroup were downloaded from GenBank. The four gene regions (ITS, nuLSU, mtSSU, and β-tubulin) were aligned using the online version of MAFFT v. 7.490. The resulting alignments were optimized and concatenated into a single 4-locus dataset using Geneious Prime. A total of 2,932 aligned characters from mitochondrial and nuclear genes were assembled into a combined matrix, representing ITS rDNA (565 bp), nuLSU rDNA (911 bp), mtSSU rDNA (692 bp), and β-tubulin (764 bp). For phylogenetic analysis, the relationships among species of Acarosporaceae were examined using Maximum likelihood (ML) and Bayesian inference (BI). Analyses were performed respectively with CIPRES Science Gateway (Miller et al. 2010) and PhyloSuite v1.2.3 (Zhang et al. 2020). Sequences of *Pycnora sorophora* were included as the outgroup. Maximum likelihood (ML) analysis was performed using RAxML-HPC v. 8.2.12 (Stamatakis 2014) on the CIPRES Science Gateway, with support values calculated from 1000 non-parametric bootstrap replicates. For the BI analysis, Partition Finder 2 (Lanfear et al. 2016) was used to determine the best-fit model for each partition, the GTR+I+G model for mtSSU, nuLSU and β-tubulin, SYM+I+G model for ITS was selected. BI analysis was conducted using MrBayes 3.2.7 (Ronquist et al. 2012). Four Markov chains were run with 2,000,000 generations for this dataset. Trees were sampled every 1000 generations, with the first 25% of trees discarded as burn-in. The consensus tree was constructed from the remaining 75% of the samples. Bootstrap support (BS) ≥ 70% and posterior probabilities (PP) ≥ 0.95 were considered significant supporting values. FigTree v. 1.4.4 (Rambaut 2016) was used to visualize the generated phylogenetic trees.

## Results and discussion

The dataset includes a total of 366 sequences (99 ITS, 96 mtSSU, 93 nuLSU, and 78 β-tubulin), of which 12 ITS, 11 mtSSU, 9 nuLSU, and 2 β-tubulin were newly generated in this study. The sequence of species with carbonized epihymenial accretions from Westberg et al. (2015) and Knudsen et al. (2025a) are included in our phylogeny.

**Figure 1.**
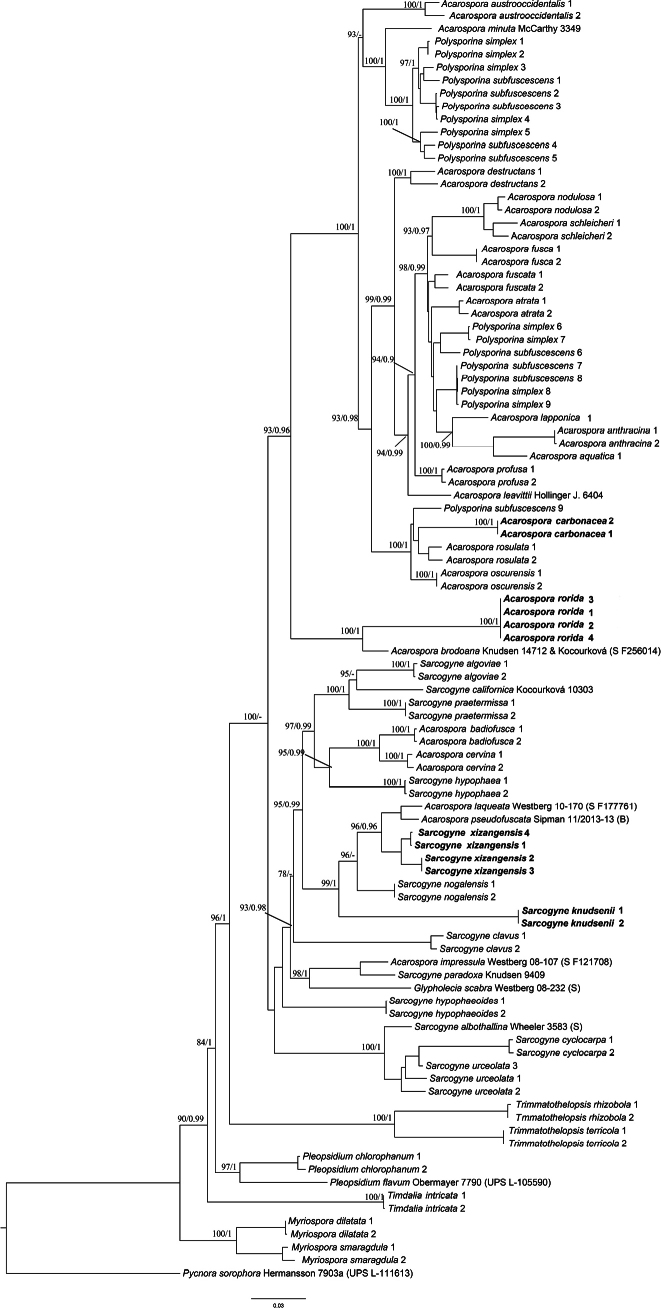
Phylogenetic tree of Acarosporaceae based on ML analysis of a four-locus combined dataset (ITS, nuLSU, mtSSU, and β-tubulin). Bootstrap support values of Maximum Likelihood (BS) ≥ 70%, and Bayesian posterior probabilities (PP) ≥ 0.95 are given near the nodes as BS/PP. Newly obtained sequences are in bold. *Pycnora sorophora* was used as an outgroup. Scale bar: 0.03 substitution per site.

Our phylogenetic tree revealed that neither *Acarospora* nor *Sarcogyne* is monophyletic (Fig. 1). Four new lineages were recovered in the phylogenetic tree, corresponding to four distinct species. Two species were recovered in *Acarospora* clade, namely *A. rorida* and *A. carbonacea*. Two species were recovered in *Sarcogyne* clade, namely *S. knudsenii* and *S. xizangensis*. The lineages of four new species were all strongly supported. The BS/PP of the lineages of *A. carbonacea* was 99/0.96, *A. rorida* was 100/1, *S. knudsenii* was 99/1, and the lineage of *S. xizangensis* was 96/0.96.

In our phylogenetic tree, *Acarospora carbonacea* was recovered in *Acarospora* clade and sister to *A. rosulata* which was found in Asia, Europe, and North America. *Acarospora carbonacea* differs from *A. rosulata* by its endolithic thallus (vs. epilithic thallus), lecideine apothecia with carbonized accretions (vs. lecanorine apothecia without carbonized accretions), and producing no secondary metabolites (vs. producing gyrophoric acid) (Knudsen et al. 2010; Güllü et al. 2023). *Acarospora rorida* was recovered at the base of the *Acarospora* clade in an isolated position. It is most closely related to *Acarospora brodoana*, which is known only from the San Bernardino Mountains in southern California. The two species were similar in having carbonized apothecia, but *A. rorida* differed in having lower hymenium 50–125 μm (vs. 125–150 μm), hyaline hypothecium (vs. black hypothecium), and it was known only from low-altitude humid areas. However, *A. brodoana* was found at elevations above 1,400 m in arid areas (Knudsen et al. 2016). *Sarcogyne xizangensis* was closely related to *A. pseudofuscata* and *A. laqueata*, but the two species differed from *S. xizangensis* by having epilithic thallus (vs. endolithic thallus), non-carbonized apothecia (vs. carbonized apothecia), and producing gyrophoric acid in *A. pseudofuscata* (vs. producing no secondary metabolites) (Sipman and Raus 2002), hymenium IKI+ blue in *A. laqueata* (vs. hymenium IKI+ blue to red) (Temina et al. 2005). In the phylogenetic tree, *S. knudsenii* was also sister to *S. xizangensis*, but differs in having wider apothecia margin 250–300 μm (vs. 50–150 μm), thinner paraphyses 2 μm (vs. 2.5–4 μm), hymenial gel IKI+ blue (vs. red or blue turning red), larger ascospores 6–7 × 3–4 μm (vs. 4–6 × 2–3 μm) and without oil drops (vs. ascospores usually with two oil drops).

### Taxonomy

#### Acarospora carbonacea

Taxon classificationFungiAcarosporalesAcarosporaceae

J.X. Wang, F.H. Liang & L. Hu sp. nov.

71421CAC-6E6B-58CC-AA4E-6633AF8C417E

863483

Fig. 2

##### Type.

China • Gansu Province: Jiuquan City, Aksay Kazakh Autonomous County, Dangjin Mountain pass, 39°19'38"N, 94°15'54"E, alt. 3719 m, on calcareous rock, 5 Aug. 2022, L. Hu et al. 20222137 (SDNU, holotype).

**Figure 2.**
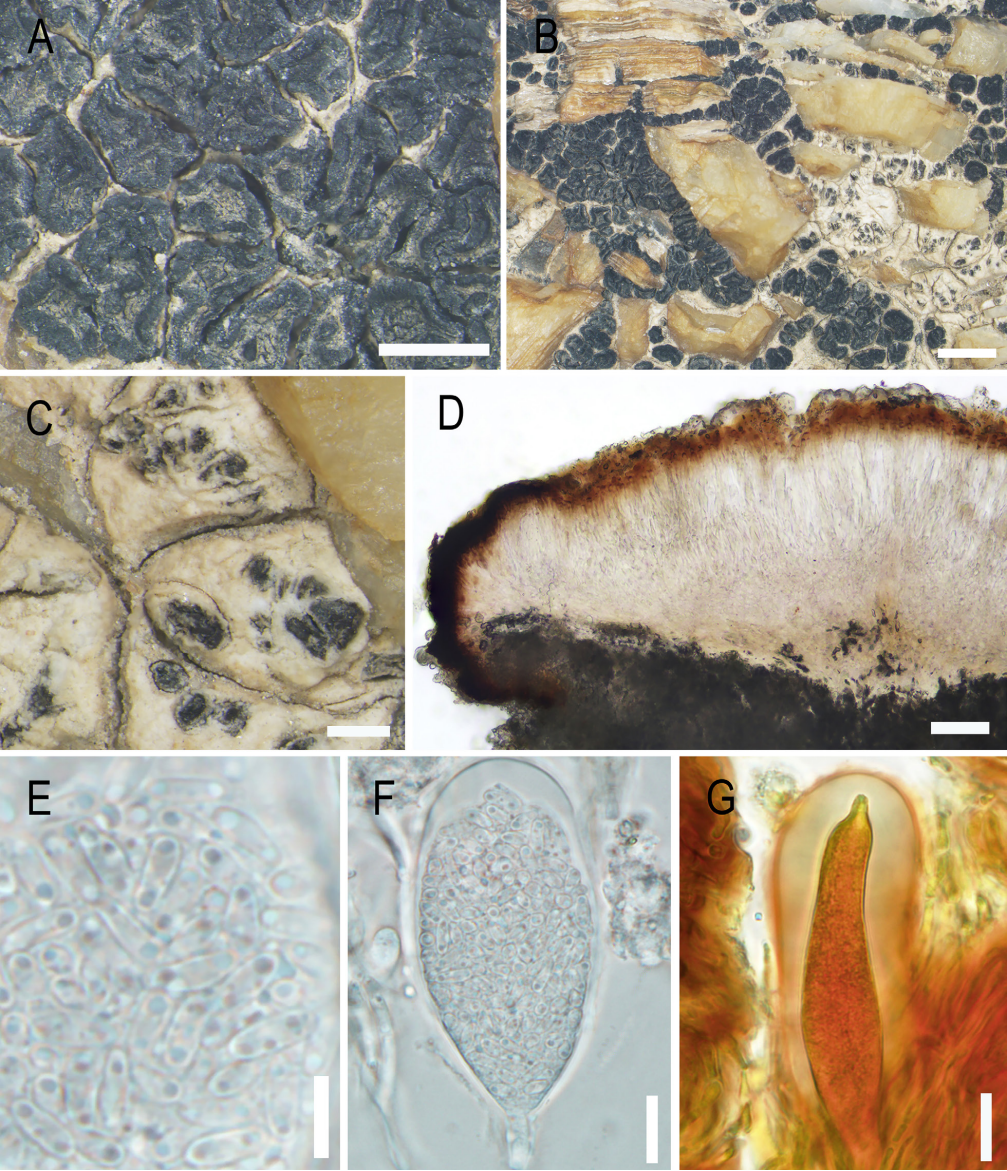
*Acarospora carbonacea*, (holotype, 20222137 SDNU). **A**. Mature apothecia; **B**. Habit of immersed thallus with black apothecia; **C**. white ecorticate chasmolithic thallus with young apothecia; **D**. Section of apothecium; **E**. Ascospores; **F**. Multispored asci; **G**. Ascus in IKI. Scale bars: 500 μm (**A**); 1 mm (**B**); 200 μm (**C**); 50 μm (**D**); 5 μm (**E**); 10 μm (**F, G**).

##### Diagnosis.

Similar to *Sarcogyne plicata* (Knudsen and Kocourková 2011) but differs by its round apothcia (vs. lirellate apothecia), disc with carbonized epihymenial accretions (vs. without carbonized epihymenial accretions), lower hymenium 60–90 μm (vs. 100–140 μm), hymenial gel IKI+ red or blue turning red (vs. IKI+ blue), and only occurs on the calcareous rocks in arid and high-altitude areas, rather than on granite in drainages, washes and flood plains.

##### Etymology.

Its name derives from the characteristic carbonization of its apothecia.

##### Description.

Thallus endolithic, algal cells 6–10 μm wide, scattered clusters in the substrate, sometimes developing white epilithic ecorticate thallus. Apothecia lecideine, round to irregular, 0.2–1 mm wide, 0.2–0.4 mm thick, usually contiguous, small apothecia usually emerging from mycelial base, sometimes compound with two hymenia, disc black usually round or irregular with carbonized epihymenial accretions, sometimes forming one umbo, slightly lower than margin. Margin segmented, usually in short linear sections at joints, 50–100 μm wide. Parathecium 40–70 μm wide, outer layer carbonized, 20–40 μm wide, inner layer hyaline, 20–30 μm wide. Hymenium 60–90 μm tall, epihymenium reddish brown, 15–25 μm tall, paraphyses 1–2 μm wide, apices expanded up to 4 μm wide in brown caps, hymenial gel IKI+ red or blue turning red, hemiamyloid. Asci clavate, 60–80 × 20–25 μm, ascospores several hundred per ascus, ellipsoid, 4–5 × 2–3 μm, usually with two oil drops. Subhymenium 40–50 μm tall, IKI+ blue to dark blue, euamyloid. Hypothecium indistinct to 10 μm thick. Pycnidia not observed. Not producing secondary metabolites.

##### Habitat and distribution.

This new species is currently known only from Jiuquan city in Gansu Province, and occurs on calcareous rocks in arid regions at an elevation of 3719 m.

##### Additional specimens examined.

China • Gansu Province: Jiuquan City, Aksay Kazakh Autonomous County, Dangjin Mountain pass, 39°19'38"N, 94°15'54"E, alt. 3719 m, on calcareous rock, 5 Aug. 2022, L. Hu et al. 20222137, 20222151 (SDNU).

##### Note.

This species is similar to *Acarospora brodoana* in having carbonized accretions on the disc surface, but is distinguished by a white thallus with epilithic and ecorticate, smaller apothecia 0.2–1 mm (vs. 1–1.5 mm), lower hymenium 60–90 μm (vs. 150–170 μm), and the absence of a black hypothecium (vs. black hypothecium) (Knudsen et al. 2016). *Acarospora carbonacea* is also similar to *A. profusa*, but the latter differs in having thinner ascus 100–90 × 10–12 μm (vs. 60–80 × 20–25 μm), smaller ascospores 1–1.5 × 1 μm (vs. 4–5 × 2–3 μm), and producing low amounts of norstictic acid (vs. producing no secondary metabolites) (Knudsen et al. 2025a).

#### Acarospora rorida

Taxon classificationFungiAcarosporalesAcarosporaceae

F.H. Liang & L. Hu sp. nov.

9A15C6EF-0860-56A7-B8EC-29CA2BC98340

863484

Fig. 3

##### Type.

China • Shandong Province: Yantai City, Muping District, Kunyu Mountain Nature Reserve, Taiboding, on the path leading down the mountain, 37°15'06"N, 121°45'36"E, alt. 752 m, on silicious rock, 22 Nov. 2024, R.T. Li et al. 20242399 (SDNU, holotype).

**Figure 3.**
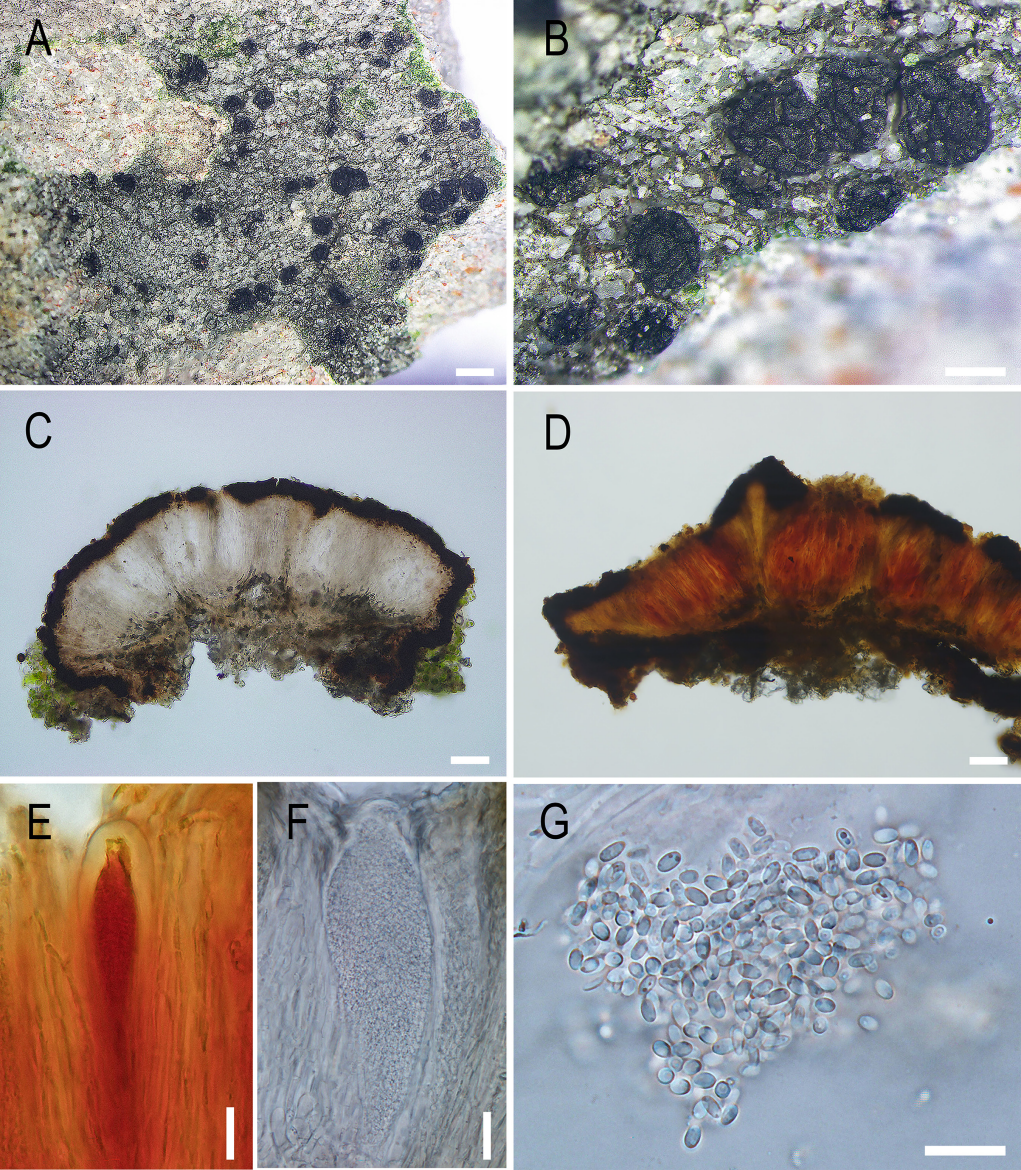
*Acarospora rorida* (holotype. 20242399 SDNU). **A**. Habit of immersed thallus with apothecia. **B**. Apothecia with carbonized accretions; **C**. Cross-section of apothecium. **D**. Hemiamyloid reaction of hymenium. **E**. *Acarospora*-type ascus. **F**. Multispored asci. **G**. Ascospores. Scale bars: 500 μm (**A**); 200 μm (**B**); 50 μm (**C, D**); 10 μm (**E–G**).

##### Diagnosis.

Similar to *Acarospora brodoana* (Knudsen et al. 2016) but differs in having smaller compound apothecia (0.25–1 mm vs. 1–1.5 mm), lower hymenium (50–125 μm vs. 150–175 μm), hypothecium hyaline (vs. black), and occurrence in humid areas at an altitude lower than 800 m (vs. at elevations above 1400 m).

##### Etymology.

The specific epithet refers to the moist habitat where this species was found.

##### Description.

Thallus endolithic. Algal layer scattered in the rock beneath the base of the apothecia or not observed, discontinuous and dispersed, algal cells globose, 8–15 μm in diam. Apothecia immersed or superficial on substrate, often dispersed, sometimes continuous, round or irregular, 0.25–1 mm in diam. Disc black, with carbonized epihymenial accretions, sometimes knobby, epruinose, rough, matt or glossy, uneven, initially flat and below margin, but then usually becoming convex and higher than the margin. Margin carbonized, usually forming a series of often irregular marginal segments. Parathecium 25–50 μm thick, with black carbonized outer layer, brown inner layer, widths variable. Hymenium 50–125 μm high, hyaline, epihymenium reddish–brown, 10–25 μm thick, with dark brown to black carbonized accretions up to 50 μm thick on the disc surface, paraphyses narrow, often 1–2 μm wide, rarely more than 2.5 μm wide, septate, infrequently branching in lower half, becoming shorter in upper third, sometimes constricted at septa, apices sometimes expanded or unexpanded in darker brown to black pigment caps, hymenium gel IKI+ usually immediately reddish orange, rarely light blue turning reddish orange, hemiamyloid. Asci clavate, 50–100 × 12–20 μm, ascospores mostly over 100 per ascus. Ascospores simple, hyaline, narrowly ellipsoid to ellipsoid, 3–4 × 1–2 μm, without oil drop. Subhymenium indistinct. Hypothecium hyaline, indistinct to 15 μm thick. Pycnidia not observed. Not producing secondary metabolites.

##### Habitat and distribution.

*Acarospora rorida* is currently known only from Shandong and Jiangsu provinces in eastern China. This new species occurs on siliceous rock at low altitudes in humid habitats, with the two collection sites separated by approximately 685 km.

##### Additional specimens examined.

China • Shandong Province: Yantai City, Muping District, Kunyu Mountain Nature Reserve, the sixth parvial field, Longxu Bridge, 37°17'33"N, 121°41'35"E, alt. 154 m, on silicious rock, 25 Nov. 2024, L.L. Zhang et al. 20242080 (SDNU); • Yantai City, Muping District, Kunyu Mountain Nature Reserve, the fifth parvial field, Huaquan Road, 37°17'38"N, 121°45'03"E, alt. 162 m, on silicious rock, 23 Nov. 2024, L.L. Zhang et al. 20242194 (SDNU); • Taiboding, on the path leading down the mountain, 37°15'06"N, 121°45'35"E, alt. 752 m, on silicious rock, 22 Nov. 2024, R.T. Li et al. 20242399 (SDNU). Jiangsu Province: Changzhou City, Liyang City, No. 96, Tianmu Lake Avenue, near Jinding Zen Temple, 31°29'35"N, 119°09'26"E, alt. 119 m, on siliceous rock, 2024, L.L. Zhang et al. 20250315 (SDNU).

##### Note.

The new species is characterized by compound apothecia with margins forming a series of irregular marginal segments, occurring at low altitudes in humid habitats. *Acarospora rorida* are morphologically similar to *Sarcogyne cyclocarpa* and *A. subfuscescens*. *Acarospora rorida* differs from *S. cyclocarpa* in having bigger ascus 50–100 × 12–20 µm (vs. 40–60 × 15–20 µm), smaller ascospores 3–4 × 1–2 µm (vs. (3.5–)4–5(–6) × (1–)2(–2.5) µm), indistinct subhymenium (vs. 40–80 µm), and growing on siliceous rocks (vs. on strongly calcareous rock) (Knudsen and Kocourková 2009; Knudsen et al. 2023b). *Acarospora rorida* differs from *A. subfuscescens* in having smaller apothecia 0.25–1 mm in diam (vs. 1–3 mm), lower hymenium 50–125 µm (vs. (80–)100–140(–180) µm), and not being parasitic on other lichens (vs. parasitic on other lichens) (Knudsen and Kocourková 2008). Furthermore, this new species with verrucose margin is similar to *Sarcogyne clavus* but differs especially in not having a dark brown hypothecium (Knudsen and Standley 2007). The segmented margin with irregular marginal segments of this species is similar to *S. hypophaea*, but that species has smooth segments usually at angles to each other (Knudsen et al. 2013). In addition, *S. paradoxa*, described from Mojave Desert in California, which we collected in northwestern China, also produces carbonized hymenial accretions. But *S. paradoxa* differs from this new species by its reddish-brown carbonized accretions (vs. black), hymenial gel euamyloid, IKI+ blue (vs. IKI+ usually immediately reddish orange, rarely light blue turning reddish orange), and smaller ascospores (3–)4–4.5(–7) × 2–3 µm (vs. 3–4 × 1–2 μm) (Knudsen and Kocourková 2020).

#### Sarcogyne knudsenii

Taxon classificationFungiAcarosporalesAcarosporaceae

J.X. Wang, F.H. Liang & L. Hu sp. nov.

C359D92D-D664-5E73-9840-D0DBD665A8DA

863487

Fig. 4

##### Type.

China • Xinjiang Uygur Autonomous Region: Tashkurgan Tajik Autonomous County, Aksay Kazakh Autonomous County, Pamir Plateau Tourist Area, 38°06'25"N, 74°59'05"E, 3544 m, on calcareous rock, 25 Jun. 2022, X.Y. Wang et al. XY22-675 (KUN, holotype).

**Figure 4.**
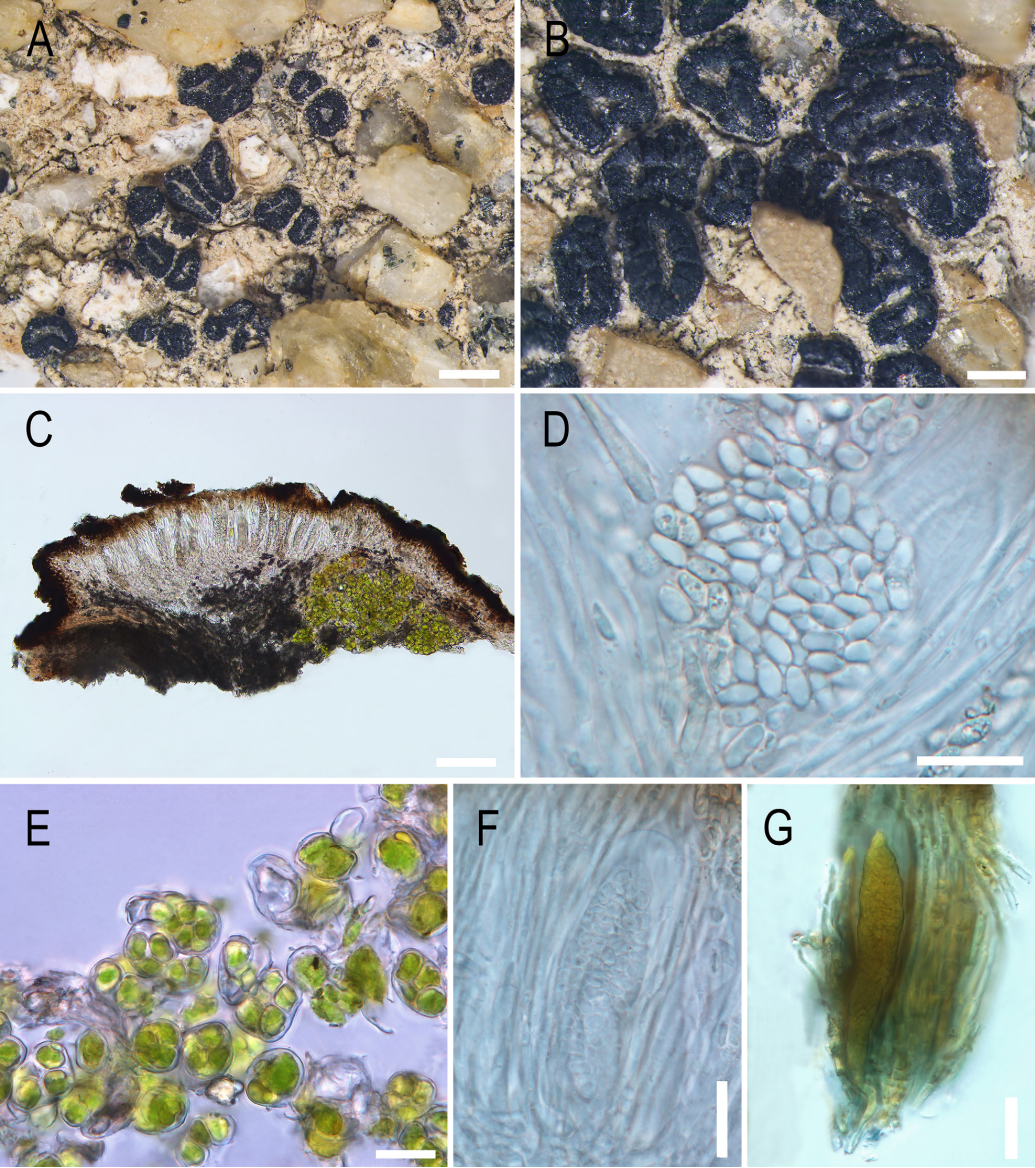
*Sarcogyne knudsenii*, (holotype, XY22-675 KUN). **A**. Habit of immersed thallus with black apothecia; **B**. Mature apothecia with segment margin; **C**. Section of apothecium; **D**. Ascospores; **E**. Algal cells; **F**. Multispored asci; **G**. Ascus in IKI. Scale bars: 500 μm (**A**); 200 mm (**B**); 50 μm (**C**); 10 μm (**D–G**).

##### Diagnosis.

Similar to the calciphyte *Sarcogyne pusilla* (Knudsen and Kocourková 2008) in its endolithic thallus and carbonized apothecia, but differs in having lower hymenium 50–100 μm (vs. 100–125 μm), larger ascospores 6–7 × 3–4 μm (vs. 4–5 × 2–3 μm), and is not parasitic on other lichen species.

##### Etymology.

This species is named in honor of the Czech lichenologist Kerry Knudsen, in recognition of his important contributions to the taxonomic and phylogenetic studies of the family Acarosporaceae in California and the southwestern United States.

##### Description.

Thallus epilithic, forming ecorticate, with scattered algal layer in the substrate or below the apothecia, occasionally absent, algal cells 6–12 μm wide. Apothecia 0.25–1 mm wide, 200 μm thick, rough and matt, immersed or superficial on rock, round to irregular, dispersed or contiguous, replicating by division. Disc black, rough, epruinose or light pruinose, concave, with carbonized accretions on surface and forming an umbo structure, sometimes disc lacking carbonized accretions. Margin carbonized, 250–300 μm thick, sometimes covering up disc, segmented, usually in long linear sections at joints, higher than the disc. Parathecium outer layer carbonized, 25–50 μm thick, inner layer light brown to hyaline, 25–75 μm thick. Hymenium 50–100 μm tall, epihymenium light brown, 10–25 μm tall, paraphyses thin, usually 1.5–2 μm wide, barely expended in dark pigment caps, hymenial gel IKI+ blue, euamyloid. Asci clavate, 60–75 × 15–20 μm, ascospores more than 100 per ascus. Ascospores ellipsoid or broadly ellipsoid, 6–7 × 3–4 μm wide, simple and hyaline, without oil drops. Subhymenium hyaline, 25–50 μm thick, IKI+ blue. Hypothecium 10–25 μm. Pycnidia not observed. Not producing secondary metabolites.

##### Habitat and distribution.

This new species is currently known only from the cold and arid Pamir Plateau in the Xinjiang Uygur Autonomous Region, and grows on calcareous rock.

##### Additional specimens examined.

China • Xinjiang Uygur Autonomous Region: Tashkurgan Tajik Autonomous County, Aksay Kazakh Autonomous County, Pamir Plateau Tourist Area, 38°06'25"N, 74°59'04"E, alt. 3573 m, on calcareous rock, 25 Jun. 2022, L.S. Wang et al. 22-71601 (KUN); • Tashkurgan Tajik Autonomous County, Aksay Kazakh Autonomous County, Pamir Plateau Tourist Area, 38°06'25"N, 74°59'05"E, 3544 m, on calcareous rock, 25 Jun. 2022, X.Y. Wang et al. XY22-675 (KUN).

##### Note.

This new species is morphologically similar to *Acarospora austrooccidentalis*, but *A. austrooccidentalis* differs in having higher hymenium (100–)120–150 μm (vs. 50–100 μm),, variable asci from 100 × 10–40 µm to 40 × 15 µm in same hymenium (vs. 60–75 × 15–20 µm), smaller ascospores (1.5–)4.0–4.6(–5.1) × (1.0–)2(–2.8) µm (vs. 6–7 × 3–4 µm), and usually occurs on non-calcareous rocks (vs. calcareous rocks), (Knudsen et al. 2025a). *Sarcogyne knudsenii* is also similar to *A. destructans* in having segmented margin of apothecia, but differs in having a hemiamyloid hymenium (vs. euamyloid hymenium), a smaller ascospores (3–)4.5(–5) × (1–)1.5–2.5 µm (vs. 6–7 × 3–4 µm), not parasitizing on other lichens, and growing on calcareous rock (vs. growing on siliceous rock) (Knudsen et al. 2022).

#### Sarcogyne xizangensis

Taxon classificationFungiAcarosporalesAcarosporaceae

F.H. Liang & L. Hu sp. nov.

8F60F397-3F3F-59F6-A285-DA4CCC738924

863486

Figs 5, 6

##### Type.

China • Xizang Autonomous Region: Nagarze County, Yamdrok Yumtso scenic area, 28°44'08"N, 91°02'19"E, alt. 4546 m, on siliceous rock, 9 Jun. 2025, Li. L. 20250906-BH118-2A (KUN, holotype).

**Figure 5.**
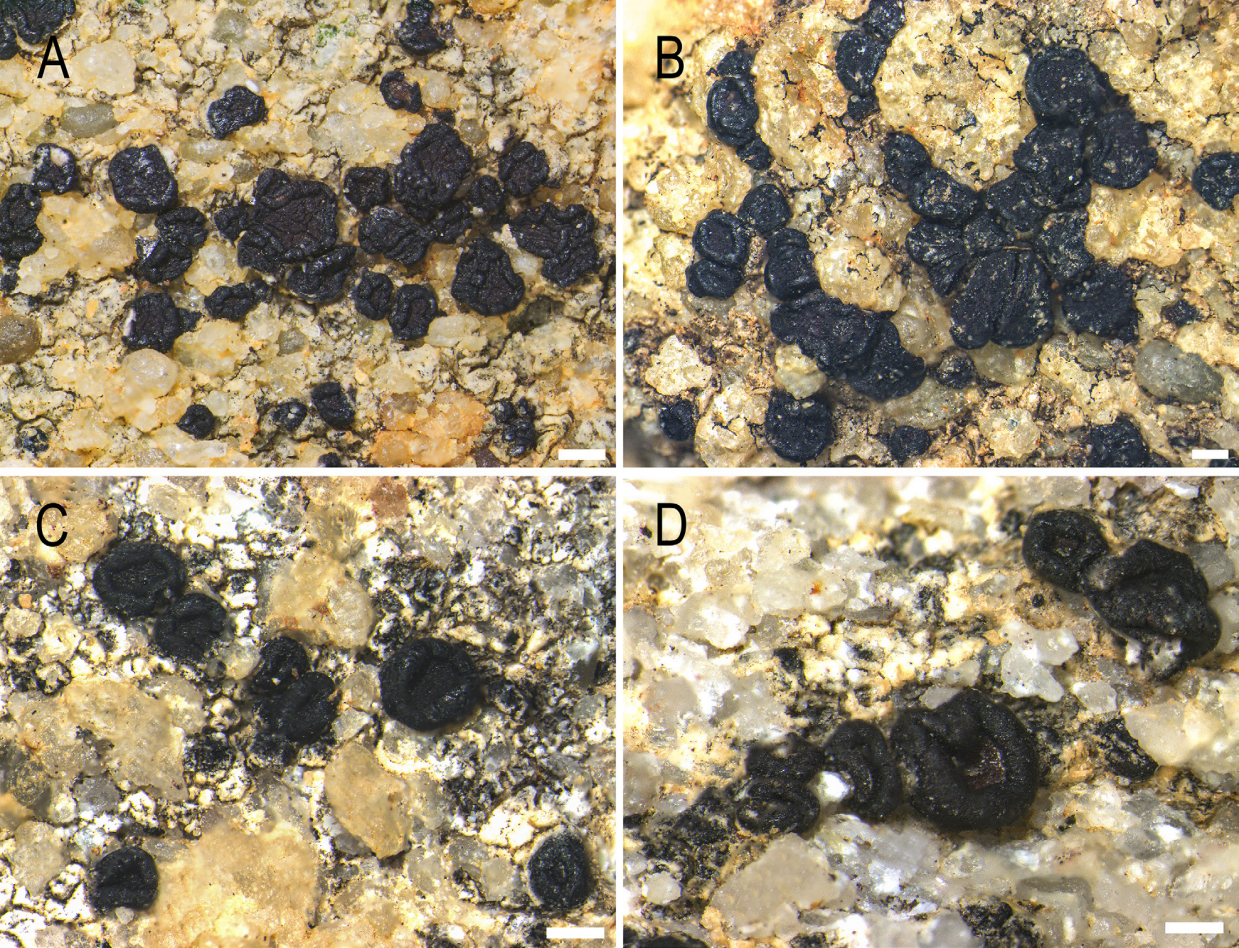
Intraspecific morphological variation of *Sarcogyne xizangensis*, **A**. Holotype, 20250906-BH118-2A KUN; **B**. 20250906-BH118-2B KUN; **C**. 20250906-BH118-5 KUN; **D**. 20250906-BH115-3 KUN. Scale bars: 200 μm (**A–D**).

**Figure 6.**
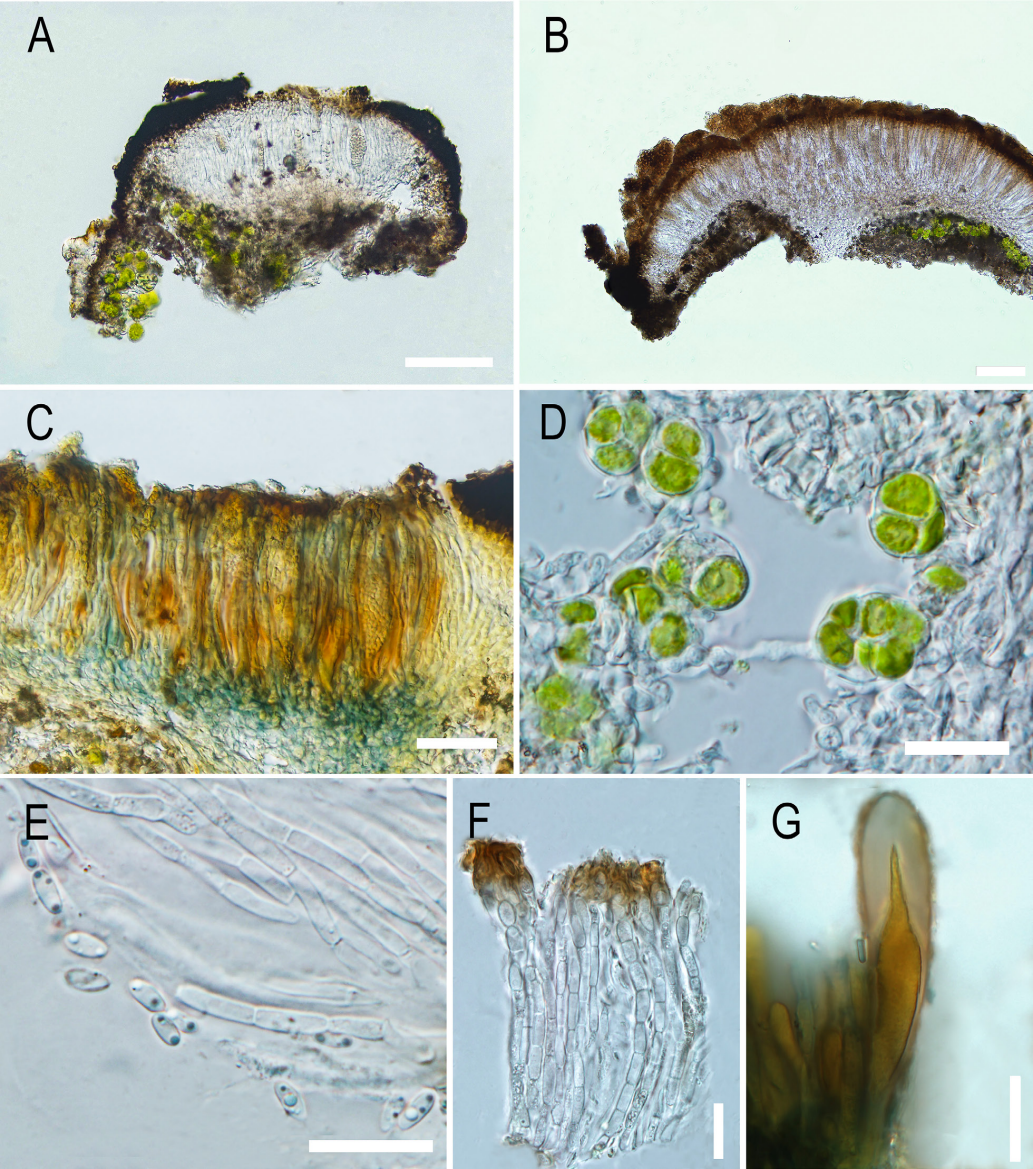
*Sarcogyne xizangensis*, (**A**. 20250906-BH118-2B KUN; **B–G**. holotype. 20250906-BH118-2A KUN). **A**. Apothecium with black carbonized accretions; **B**. Apothecium with brown accretions; **C**. Hemiamyloid reaction of hymenium; **D**. Algal cells; **E**. Ascospores; **F**. Paraphyses; **G**. Ascus in IKI. Scale bars: 50 μm (**A**); 20 mm (**B, C**); 10 μm (**D–G**).

##### Diagnosis.

Similar to *Acarospora privigna* (Kantvilas 1998; Knudsen et al. 2021b), but differs in having lower hymenium (60–95 μm vs. 90–130 μm), wider paraphyses (2.5–4 μm vs. 1–1.5 μm), and ellipsoid to broadly ellipsoid ascospores (vs. bacilliform to narrowly ellipsoidal).

##### Etymology.

The specific epithet is derived from the type locality, Xizang Autonomous Region (Tibet), where this species was collected.

##### Description.

Thallus endolithic, algae usually in clusters in substrate or at base of apothecium, algae cells 6–12 μm wide. Apothecia black, light shiny, 0.1–0.75 mm wide, 250–300 μm thick, dispersed or forming conspicuous clusters through replication, becoming compound apothecia, young apothecia usually round, without carbonized epihymenium accretions, and usually becoming rough with gobs of melanin build-up on the disc, but sometimes just smooth and only the margin carbonized. Disc dark reddish brown to black, red when wetted, epruinose, flat and below the margin, sometimes on an equal level with margin, rarely convex. Margin carbonized, 150 μm thick when young, 50 μm thick when mature, usually raised above the disc, partly knobby or crenulate, sometimes entire, not becoming segmented. Parathecium 20–60 μm thick, out layer black, 10–35 μm thick, inner brown, 10–25 μm thick. Hymenium 60–95 μm tall, epihymenium reddish–brown, 10–30 μm tall, occasionally with brown or black carbonized accretions, sometimes lack accretions, paraphyses 2.5–4 μm wide, infrequent branch, light expand to 5 μm wide in pigment caps, hymenial gel IKI+ red or blue turning red, hemiamyloid. Asci 50–70 × 12–20 μm, hundreds of ascospores, 4–6 × 2–3 μm wide, ellipsoid to broadly ellipsoid, simple and hyaline, usually with two oil drops. Subhymenium 25–50 μm thick, IKI+ blue. Hypothecium indistinct to 10 μm thick. Pycnidia not observed. Not producing secondary metabolites.

##### Habitat and distribution.

This species is only found in the high-altitude areas of the Qinghai-Xizang Plateau in western China, growing on calcareous or siliceous rocks.

##### Additional specimens examined.

China • Xizang Autonomous Region: Nagarze County, Yamdrok Yumtso scenic area, 28°44'08"N, 91°02'19"E, alt. 4546 m, on siliceous rock, 9 Jun. 2025, Li.L. 20250906-BH118-2A, 20250906-BH118-2B (KUN); • Nagarze County, Yamdrok Yumtso scenic area, 28°44'57"N, 90°53'18"E, alt. 4545 m, on siliceous rock, 9 Jun. 2025, Li.L. 20250906-BH115-3 (KUN); • Nagarze County, Yamdrok Yumtso scenic area, 28°44'08"N, 91°02'19"E, alt. 4547 m, on calcareous rock, 9 Jun. 2025, Li.L. 20250906-BH118-5 (KUN).

##### Note.

This new species has wider paraphyses 2.5–4 μm, which can easily distinguish from other *Sarcogyne* species. In addition, the new species has variable morphological characteristics. The specimen (20250906-BH118-2A KUN) has obviously segmented margin, and discs with carbonized epihymenial acceretions, while the other specimens have entire and smooth margin, rarely becoming segmented, and discs usually observe no carbonized epihymenial accretions.

*Sarcogyne xizangensis* is similar to *S. albothallina* by its carbonized apothecia, but it differs from *S. albothallina* by endolithic thallus (vs. epilithic thallus), wider paraphyses 2.5–4 μm (vs. 1.5–2.5 μm), smaller ascospores 4–6 × 2–3 μm (vs. 6.0–7.5 × 3–4 μm), and not producing secondary metabolites (vs. producing 4-O-methylhiascic acid) (Knudsen et al. 2016). *Sarcogyne xizangensis* differs from *S. pakistanensis* in wider paraphyses 2.5–4 μm (vs. 2–3 μm), larger ascospores 4–6 × 2–3 μm (vs. 3–4 × 1–2 μm), hymenial gel IKI+ red or blue turning red (vs. IKI+ dark blue), in not producing secondary metabolites (vs. 2'-O-demethylpsoromic acid, psoromic acid detected), and in producing carbonized epihymenial accretions (vs. without carbonized epihymenial accretions) (Fayyaz et al. 2023).

## Supplementary Material

XML Treatment for Acarospora carbonacea

XML Treatment for Acarospora rorida

XML Treatment for Sarcogyne knudsenii

XML Treatment for Sarcogyne xizangensis

## Additional information

### Artificial Intelligence (AI) use

The authors accept full responsibility for the content of the manuscript, including the disclosure of any use of AI.

No AI tools were used in the preparation of this manuscript.

### Author ORCIDs

F.H. Liang https://orcid.org/0009-0000-7210-0607

J.X. Wang https://orcid.org/0009-0008-6376-4071

M. Ai https://orcid.org/0000-0002-7753-3310

W. Jiang https://orcid.org/0009-0006-4481-2774

S.N. Zhou https://orcid.org/0009-0008-4568-6639

Z.T. Zhao https://orcid.org/0009-0007-2970-9231

X.Y. Wang https://orcid.org/0000-0003-2166-6111

L. Hu https://orcid.org/0000-0002-9451-383X

### Data availability

All of the data that support the findings of this study are available in the main text or Supplementary Information.

## Supplementary materials

Supplementary material 1 A list of sampled specimens included in the molecular phylogeny Data type docx https://binary.pensoft.net/file/1673244This dataset is made available under the Open Database License (http://opendatacommons.org/licenses/odbl/1.0/). The Open Database License (ODbL) is a license agreement intended to allow users to freely share, modify, and use this Dataset while maintaining this same freedom for others, provided that the original source and author(s) are credited.Author: Fuhui Liang

Supplementary material 2 Phylogenetic tree constructed through BI analyses based on ITS, nuLSU, mtSSU, and β-tubulin for Acarosporaceae Data type docx Explanation note *Pycnora sorophora* was used as the outgroup. Only significant values (higher than 0.95 PP) are shown https://binary.pensoft.net/file/1673245This dataset is made available under the Open Database License (http://opendatacommons.org/licenses/odbl/1.0/). The Open Database License (ODbL) is a license agreement intended to allow users to freely share, modify, and use this Dataset while maintaining this same freedom for others, provided that the original source and author(s) are credited.Author: Fuhui Liang
