# Supplementary material for: Four new species of Acarosporaceae (Acarosporales, Lecanoromycetes) with carbonized epihymenial accretions from China
Source: MycoKeys. 2026 Jun 8;133:367–85. doi: 10.3897/mycokeys.133.196437 (PMC13270225; doi:10.3897/mycokeys.133.196437)
Supplement: Supplementary material 1 — A list of sampled specimens included in the molecular phylogeny [file mycokeys-133-367-s001.docx]

**Supplementary material 1**

Table: **Specimen details (country, voucher, GenBank accession) are provided for all samples included in the molecular phylogeny; newly obtained sequences are in bold.**

| **Species** | **Country** | **Voucher** | **ITS** | **mtSSU** | **nuLSU** | **β-TUB** |
| --- | --- | --- | --- | --- | --- | --- |
| *Acarospora anthracina* 1 | USA | BRY C25453 | PQ146128 | PQ149099 | PQ186803 | PQ280745 |
| *A. anthracina* 2 | USA | Hollinger J. 13181 | PQ146134 | PQ149105 | PQ186809 | PQ280747 |
| *A. aquatica* | USA | Lendemer 11526 | PQ146145 | PQ149115 | PQ186820 | PQ317751 |
| *A. atrata* 1 | Sweden | Arup L02737 (LD) | LN810760 | LN810885 | LN810760 | LN810652 |
| *A. atrata* 2 | Norway | Westberg 08-125 (S F124797) | LN810761 | LN810886 | LN810761 | LN810653 |
| *A. austrooccidentalis* 1 | USA | Kocourkova 10842 | OQ171068 | OQ184780 | OQ195833 | PQ317752 |
| *A. austrooccidentalis* 2 | USA | Kocourkova 10842.2 | OQ171083 | OQ184795 | OQ195846 | - |
| *A. badiofusca* 1 | Sweden | Nordin 5552 (UPS L-124833) | LN810762 | LN810887 | LN810762 | LN810654 |
| *A. badiofusca* 2 | Sweden | Nordin & Owe-Larsson 36 (UPS) | LN810763 | LN810888 | LN810763 | LN810655 |
| *A. brodoana* | USA | Knudsen 14712 & Kocourková (S F256014) | LN810882 | LN810955 | LN810882 | - |
| ***A. carbonacea* 1** | **China** | **SDNU20222152** | **PZ162617** | **PZ162631** | **PZ162623** | **-** |
| ***A. carbonacea* 2** | **China** | **SDNU20222137** | **PZ162616** | **PZ162630** | **PZ162622** | **-** |
| *A. cervina* 1 | Switzerland | Westberg 10-172 (S F177758) | LN810764 | LN810889 | LN810764 | LN810656 |
| *A. cervina* 2 | Sweden | Westberg SAR200 (S) | LN810765 | LN810890 | LN810765 | LN810657 |
| *A. destructans* 1 | USA | Dart 1490 | PQ146129 | - | PQ186804 | PQ399796 |
| *A. destructans* 2 | USA | Hollinger J. 16408 | PQ146130 | PQ149101 | PQ186805 | PQ368717 |
| *A. fusca* 1 | Sweden | Westberg 10-106 (S) | LN810758 | LN810883 | LN810758 | LN810650 |
| *A. fusca* 2 | Sweden | Westberg 10-108 (S) | LN810759 | LN810884 | LN810759 | LN810651 |
| *A. fuscata* 1 | Sweden | Westberg SAR120 (LD) | LN810766 | LN810891 | LN810766 | LN810658 |
| *A. fuscata* 2 | Sweden | Westberg SAR129 (LD) | LN810767 | LN810892 | LN810767 | LN810659 |
| *A. impressula* | Norway | Westberg 08-107 (S F121708) | LN810776 | LN810901 | LN810776 | LN810668 |
| *A. lapponica* | Czech Republic | Bouda (923411 PRM) | OP162380 | OP177764 | OP216698 | PQ204640 |
| *A. laqueata* | Switzerland | Westberg 10-170 (S F177761) | LN810778 | LN810903 | LN810778 | LN810670 |
| *A. leavittii* | USA | Hollinger J. 6404 | PQ146139 | PQ149109 | PQ186814 | - |
| *A. minuta* | Canada | McCarthy 3349 | ON707076 | ON715672 | ON725167 | PQ399794 |
| *A. nodulosa* 1 | Spain | Westberg 10-215 (S) | LN810789 | LN810914 | LN810789 | LN810681 |
| *A. nodulosa* 2 | Spain | Westberg SCIN032 (S) | LN810788 | LN810913 | LN810788 | LN810680 |
| *A. oscurensis* 1 | USA | Kocourkova 10795 | OQ171046 | OQ184762 | OQ195817 | PQ368712 |
| *A. oscurensis* 2 | USA | Kocourkova 10795.2 | OQ171047 | OQ184763 | OQ195818 | PQ256829 |
| *A. profusa* 1 | USA | Leavitt S. 23187 | PQ146125 | PQ149096 |  | PQ399798 |
| *A. profusa* 2 | USA | Leavitt S. 23203 | PQ146126 | PQ149097 | PQ186801 |  |
| *A. pseudofuscata* | Greece | Sipman 11/2013-13 (B) | MZ262718 | MZ262730 | MZ262741 | - |
| *A. rosulata* 1 | USA | Knudsen 9509 (S F256011) | LN810796 | LN810921 | LN810796 | LN810688 |
| *A. rosulata* 2 | Norway | Westberg 08-193 (S) | LN810797 | LN810922 | LN810797 | LN810689 |
| *A. schleicheri* 1 | China | Obermayer 2919 (UPS L-070426) | LN810800 | LN810925 | LN810800 | LN810692 |
| *A. schleicheri* 2 | Arizona | Sweat KGS1196 (UPS) | LN810801 | LN810926 | LN810801 | LN810693 |
| ***A. rorida* 1** | **China** | **SDNU20250313** | **PV989554** | **PV989561** | **PV989566** | **-** |
| ***A. rorida* 2** | **China** | **SDNU20242399** | **PV989555** | **PV989563** | **PV989568** | **-** |
| ***A. rorida* 3** | **China** | **SDNU20242080** | **PV989556** | **PV989562** | **PV989567** | **-** |
| ***A. rorida* 4** | **China** | **SDNY20242240** | **PV989557** | **-** | **-** | **-** |
| *Glypholecia scabra* | Norway | Westberg 08-232 (S) | LN810811 | LN810936 | LN810811 | LN810703 |
| *Myriospora dilatata* 1 | Sweden | Nordin 5507 (UPS L-124304) | EU870660 | EU870712 | LN810871 | EU870770 |
| *M. dilatata* 2 | Sweden | Baloch SW116 (S F114109, holotype) | EU870656 | EU870708 | LN810872 | EU870766 |
| *M. smaragdula* 1 | Sweden | Ågren 384 (UPS L-098484) | EU870686 | EU870738 | LN810878 | EU870798 |
| *M. smaragdula* 2 | Sweden | Wedin 6620 (UPS) | EU870688 | EU870740 | LN810879 | EU870800 |
| *Pleopsidium chlorophanum* 1 | Sweden | Nordin 4439 (UPS L-076485) | EU870691 | EU870743 | EU870691 | EU870805 |
| *P. chlorophanum* 2 | Sweden | Nordin 6209 (UPS L-179248) | LN810813 | LN810938 | LN810813 | LN810705 |
| *P. flavum* | Austria | Obermayer 7790 (UPS L-105590) | AY853385 | AY853336 | AY853385 | EU870806 |
| *Polysporina simplex* 1 | Norway | Westberg 08-270 (S F122563) | LN810823 | LN810948 | LN810823 | LN810713 |
| *P. simplex* 2 | Austria | Westberg SAR273 (S) | LN810826 | LN810951 | LN810826 | LN810716 |
| *P. simplex* 3 | Sweden | Westberg P118 (S) | LN810825 | LN810950 | LN810825 | LN810715 |
| *P. simplex* 4 | Norway | Westberg 08-258 (S F122602) | LN810819 | LN810944 | LN810819 | LN810710 |
| *P. simplex* 5 | Norway | Westberg 08-247 (S F122590) | LN810824 | LN810949 | LN810824 | LN810714 |
| *P. simplex* 6 | Sweden | Westberg 09-455 (S F152846) | LN810820 | LN810945 | LN810820 | LN810711 |
| *P. simplex* 7 | Sweden | Westberg SAR199 (S) | LN810821 | LN810946 | LN810821 | LN810712 |
| *P. simplex* 8 | Sweden | Westberg 06-020 (LD 1267752) | LN810827 | LN810952 | LN810827 | LN810717 |
| *P. simplex* 9 | Norway | Westberg 08-134 (S F123693) | LN810818 | LN810943 | LN810818 | LN810709 |
| *P. subfuscescens* 1 | Norway | Westberg 08-281 (S F122560) | LN810830 | LN810956 | LN810830 | LN810719 |
| *P. subfuscescens* 2 | Sweden | Westberg 09-566 (S) | LN810838 | LN810964 | LN810838 | LN810727 |
| *P. subfuscescens* 3 | Norway | Westberg 08-154 (S F152849) | LN810832 | LN810958 | LN810832 | LN810721 |
| *P. subfuscescens* 4 | Norway | Westberg 08-240 (S) | LN810836 | LN810962 | LN810836 | LN810725 |
| *P. subfuscescens* 5 | Sweden | Westberg 09-638 (S) | LN810837 | LN810963 | LN810837 | LN810726 |
| *P. subfuscescens* 6 | Sweden | Westberg & Westberg 06-118 (LD 1264167) | LN810848 | LN810974 | LN810848 | LN810737 |
| *P. subfuscescens* 7 | Norway | Westberg 08-136 (S F123694) | LN810833 | LN810959 | LN810833 | LN810722 |
| *P. subfuscescens* 8 | Sweden | Westberg 09-169 (S F138167) | LN810831 | LN810957 | LN810831 | LN810720 |
| *P. subfuscescens* 9 | USA | Knudsen 9405 (S) | LN810847 | LN810973 | LN810847 | LN810736 |
| *Pycnora sorophora* | Sweden | Hermansson 7903a (UPS L-111613) | FJ959357 | AY853338 | AY853387 | LN810757 |
| *Sarcogyne. albothallina* | USA | Wheeler 3583 (S) | LN810829 | LN810954 | LN810829 | LN810718 |
| *S. algoviae* 1 | Norway | Westberg 08-276 (S F122564) | LN810849 | LN810975 | LN810849 | LN810738 |
| *S. algoviae* 2 | Norway | Westberg 08-168 (S F122537) | LN810850 | LN810976 | LN810850 | LN810739 |
| *S. californica* | USA | Kocourková 10303 | OK142769 | OK032154 | - | - |
| *S. clavus* 1 | Austria | Obermayer 09129 (GZU 49-2002) | LN810852 | LN810978 | LN810852 | LN810741 |
| *S. clavus* 2 | Sweden | Berglund SAR220 (S) | LN810853 | - | LN810853 | LN810742 |
| *S. cyclocarpa* 1 | Sweden | Westberg P117 (S) | LN810815 | LN810940 | LN810815 | - |
| *S. cyclocarpa* 2 | Norway | Westberg 08-265 (S F123674) | LN810816 | LN810941 | LN810816 | - |
| *S. hypophaea* 1 | Sweden | Westberg SAR198 (S) | LN810856 | LN810981 | LN810856 | LN810745 |
| *S. hypophaea* 2 | Finland | Pykälä 23561 (H) | LN810857 | LN810982 | LN810857 | LN810746 |
| *S. hypophaeoides* 1 | Sweden | Westberg 08-002 (S F119718) | LN810858 | LN810983 | LN810858 | LN810747 |
| *S. hypophaeoides* 2 | Norway | Westberg 08-139 (S F123697) | LN810859 | LN810984 | LN810859 | LN810748 |
| ***S. knudsenii* 1** | **China** | **KUN-XY22-675** | **PZ162615** | **PZ162629** | **PZ162621** | **√** |
| ***S. knudsenii* 2** | **China** | **KUN-22-71601** | **PZ162610** | **PZ162624** | **-** | **√** |
| *S. nogalensis* 1 | New Mexico | Knudsen 19340 (SBBG) | ON447632 | ON367905 | ON391460 | - |
| *S. nogalensis* 2 | USA | Knudsen 19340 (BRY-C) | OK142762 | OK032147 | ON391460 | - |
| *S. paradoxa* | USA | Knudsen 9409 | LN810814 | LN810939 | LN810814 | LN810706 |
| *S. praetermissa* 1 | Finland | Pykälä 22279 (H) | LN810862 | LN810987 | LN810862 | - |
| *S. praetermissa* 2 | Finland | Pykälä 28542 (H) | LN810863 | LN810988 | LN810863 | - |
| *S. urceolata* 1 | Slovakia | Westberg s.n. (UPS L-1075710) | PQ249835 | PQ249831 | PQ249835 | PQ399800 |
| *S. urceolata* 2 | Norway | Klepsland s.n. (UPS L-926140) | PQ249836 | PQ249832 | PQ249836 | PQ399799 |
| *S. urceolata* 3 | Norway | Westberg 08-260 (S F123679) | LN810840 | LN810966 | LN810816 | LN810729 |
| ***S. xizangensis* 1** | **China** | **KUN-20250906-BH118-2B** | **PZ162613** | **PZ162627** | **PZ162620** | **PZ233673** |
| ***S. xizangensis* 2** | **China** | **KUN-20250906-BH115-3** | **PZ162611** | **PZ162625** | **PZ162618** | **-** |
| ***S. xizangensis* 3** | **China** | **KUN-20250906-BH118-5** | **PZ162614** | **PZ162628** | **-** | **-** |
| ***S. xizangensis* 4** | **China** | **KUN-20250906-BH118-2A** | **PZ162612** | **PZ162626** | **PZ162619** | **PZ233672** |
| *Timdalia intricata* 1 | Sweden | Westberg P114 (S) | LN810867 | LN810992 | LN810867 | LN810756 |
| *T. intricata* 2 | Sweden | Westberg SAR92 (LD) | LN810866 | LN810991 | LN810866 | LN810755 |
| *Trimmatothelopsis rhizobola* 1 | Sweden | Westberg 2994 (LD) | EU870640 | EU870692 | LN810868 | EU870745 |
| *T. rhizobola* 2 | Sweden | Westberg 3099 (LD) | EU870641 | EU870693 | LN810869 | EU870746 |
| *T. terricola* 1 | USA | Knudsen 11216 & Sagar (S F256012) | LN810806 | LN810931 | LN810806 | LN810698 |
| *T. terricola* 2 | USA | Knudsen 11216 & Sagar (S F256013) | LN810807 | LN810932 | LN810807 | LN810699 |
