# Supplementary figures and images for: Four new species of Acarosporaceae (Acarosporales, Lecanoromycetes) with carbonized epihymenial accretions from China
Source: MycoKeys. 2026 Jun 8;133:367–85. doi: 10.3897/mycokeys.133.196437 (PMC13270225; doi:10.3897/mycokeys.133.196437)

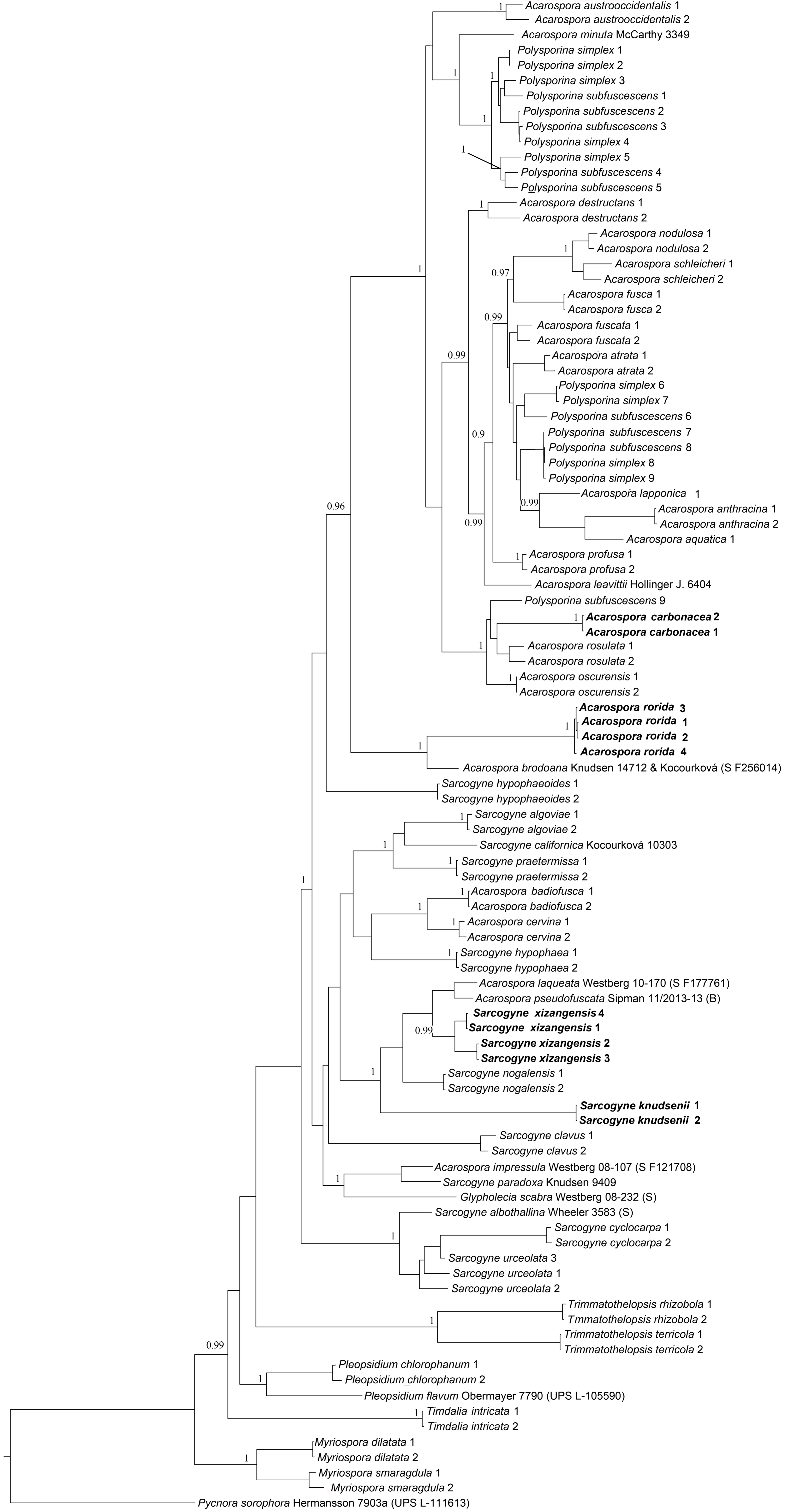

0.03

Supplement: Supplementary material 2 — Phylogenetic tree constructed through BI analyses based on ITS, nuLSU, mtSSU, and β-tubulin for Acarosporaceae [file mycokeys-133-367-s002.pdf]
